# Positive single-center randomized trials and subsequent multicenter randomized trials in critically ill patients: a systematic review

**DOI:** 10.1186/s13054-023-04755-5

**Published:** 2023-11-28

**Authors:** Yuki Kotani, Stefano Turi, Alessandro Ortalda, Martina Baiardo Redaelli, Cristiano Marchetti, Giovanni Landoni, Rinaldo Bellomo

**Affiliations:** 1 Department of Anesthesia and Intensive Care, IRCCS San Raffaele Scientific Institute, Via Olgettina 60, 20132 Milan, Italy; 2 School of Medicine, Vita-Salute San Raffaele University, Via Olgettina 58, 20132 Milan, Italy; 3 Department of Intensive Care Medicine, Kameda Medical Center, 929 Higashi-cho, Kamogawa, Chiba 296-8602 Japan; 4 Department of Critical Care, The University of Melbourne, Melbourne, Australia; 5 Australian and New Zealand Intensive Care Research Centre, Monash University, Melbourne, Australia

**Keywords:** Intensive care units, Critical illness, Mortality, Randomized controlled trial, Guideline, Systematic review

## Abstract

**Background:**

It is unclear how often survival benefits observed in single-center randomized controlled trials (sRCTs) involving critically ill patients are confirmed by subsequent multicenter randomized controlled trials (mRCTs). We aimed to perform a systemic literature review of sRCTs with a statistically significant mortality reduction and to evaluate whether subsequent mRCTs confirmed such reduction.

**Methods:**

We searched PubMed for sRCTs published in the New England Journal of Medicine, JAMA, or Lancet, from inception until December 31, 2016. We selected studies reporting a statistically significant mortality decrease using any intervention (drug, technique, or strategy) in adult critically ill patients. We then searched for subsequent mRCTs addressing the same research question tested by the sRCT. We compared the concordance of results between sRCTs and mRCTs when any mRCT was available. We registered this systematic review in the PROSPERO International Prospective Register of Systematic Reviews (CRD42023455362).

**Results:**

We identified 19 sRCTs reporting a significant mortality reduction in adult critically ill patients. For 16 sRCTs, we identified at least one subsequent mRCT (24 trials in total), while the interventions from three sRCTs have not yet been addressed in a subsequent mRCT. Only one out of 16 sRCTs (6%) was followed by a mRCT replicating a significant mortality reduction; 14 (88%) were followed by mRCTs with no mortality difference. The positive finding of one sRCT (6%) on intensive glycemic control was contradicted by a subsequent mRCT showing a significant mortality increase. Of the 14 sRCTs referenced at least once in international guidelines, six (43%) have since been either removed or suggested against in the most recent versions of relevant guidelines.

**Conclusion:**

Mortality reduction shown by sRCTs is typically not replicated by mRCTs. The findings of sRCTs should be considered hypothesis-generating and should not contribute to guidelines.

**Supplementary Information:**

The online version contains supplementary material available at 10.1186/s13054-023-04755-5.

## Background

Randomized controlled trials (RCTs) are widely accepted as the best available tools to provide scientific evidence, are considered integral to informed clinical decision-making [1] and have been and remain the gold standard for assessing the efficacy of therapeutic agents. However, despite their potential to generate robust evidence, the positive results of single-center randomized controlled trials (sRCTs) may not be replicated when subjected to large multicenter randomized controlled trials (mRCTs), particularly within the context of intensive care settings [2, 3]. The discrepancies in results are often attributed to the inherent limitations of sRCTs. These limitations typically include biases due to local effects, minimal heterogeneity among the enrolled patients, inadequate blinding of personnel and data analysis, and the temporal gap between enrollment completion and publication. In addition, many sRCTs conducted in intensive care settings are often characterized by a low fragility index, indicating that the positive findings of the study depend on a small number of events [4]. Therefore, clinicians should interpret the positive evidence from sRCTs with caution, as clinical practice based on such evidence carries a high risk of bias [2].

Despite the above considerations, no study has systematically evaluated the discrepancy between positive sRCTs and subsequent mRCTs in the intensive care setting to provide a detailed perspective on the reproducibly of sRCTs. Therefore, we conducted a systematic review to identify sRCTs showing a mortality increase or decrease with a statistically significant difference and to evaluate whether following mRCTs confirmed or refuted the positive findings of these sRCTs. The primary objective of this systematic review was to report if significant mortality reduction observed in sRCTs was replicated in subsequent mRCTs. The secondary objective was to observe how clinical guidelines have dealt with these positive sRCTs in their recommendations.

## Methods

We performed a systematic review according to the Preferred Reporting Items for Systematic Reviews and Meta-Analyses (PRISMA) guidelines [5] (see PRISMA checklist in Additional file 1). This systematic review was registered in PROSPERO International Prospective Register of Systematic Reviews (CRD42023455362).

### Search strategy and selection criteria

Two investigators independently searched PubMed for all RCTs of any non-surgical intervention influencing unadjusted landmark mortality in critically ill patients (> 48 h after randomization), published in three medical journals (i.e., New England Journal of Medicine, JAMA, and Lancet) from inception to December 31st, 2016. We did not consider sRCTs published after 2017 considering the time lag between the publication of sRCTs and their corresponding mRCTs.

We considered a difference in mortality as statistically significant when present at a specific point (> 48 h after randomization) with simple statistical tests and without adjustment for baseline characteristics. We selected articles published in NEJM, JAMA, or the Lancet, with a randomized controlled trial design in a single-center setting, presenting a statistically significant reduction or increase in unadjusted landmark mortality in critically ill patients. A quasi-randomized or non-randomized methodology, multicentric trials, pediatric populations, and absence of data on mortality were considered exclusion criteria. The full PubMed search strategy is available in Additional file 1.

After identification of eligible sRCTs, two investigators independently searched for mRCTs addressing the same PICO (population, intervention, control, outcome) frameworks, which were published from inception to December 31st, 2022.

The risk of bias of each included sRCT was assessed using the Cochrane risk-of-bias tool for randomized trials version 2 (RoB 2) [6].

### Data extraction

Two investigators extracted the following variables in a standardized data collection form: PubMed unique identifier, journal, first author, year of publication, study population, number of patients enrolled, intervention, control, mortality data with statistical significance, and timepoint of mortality assessment. If a subsequent mRCT was identified, we evaluated whether the mortality findings of the mRCT were consistent with those of the sRCT. Furthermore, we explored whether sRCTs were incorporated into international clinical practice guidelines. We further assessed whether and when guidelines stopped citing such RCTs or issued recommendations modified by the mRCTs findings.

### Statistical analysis

First, positive sRCTs with at least one subsequent mRCT were classified into three groups based on the results of mRCTs: significant mortality reduction (positive mRCTs), no significant difference in mortality (neutral mRCTs), and significant mortality increase (negative mRCTs). The proportion of sRCTs within each group was reported accordingly.

Second, we categorized included sRCTs that were cited at least once in international clinical guidelines based on the current guideline recommendations: supporting the intervention shown to have survival benefits in the sRCT, withholding recommendation due to insufficient evidence, and opposing the intervention of interest or excluding the sRCT cited in the preceding version of the guidelines.

To confirm the robustness of our findings, we performed a sensitivity analysis including only recent positive sRCTs published after 2001 to describe the mortality results of subsequent mRCTs and the guideline recommendations regarding the intervention assessed in the included sRCTs.

Furthermore, the following data were summarized: the number of randomized patients (sRCTs and mRCTs), the number of participating centers (mRCTs), the duration between publications of the sRCT and subsequent mRCT, and the duration from the initial citation of the sRCTs in the guidelines to an alteration in recommendation against its use or removal from guidelines. Missing data were not imputed throughout this study. Continuous variables were described as median and interquartile range (IQR). Categorical variables were expressed as number (percentage). We used RStudio Version 2023.06.0+421 (RStudio Team, Boston, United States).

## Results

**Fig. 1:**
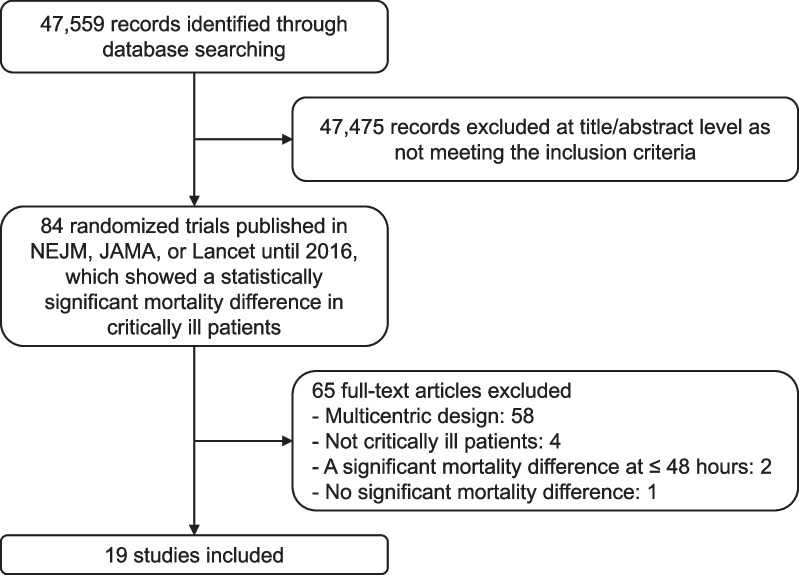
Flow chart of study selection. *NEJM* New England Journal of Medicine

We identified 19 sRCTs published in the three high impact factor journals (7 in New England Journal of Medicine, 7 in JAMA, or 5 in Lancet), which showed a statistically significant mortality difference in critically ill patients [7–25] (Fig. 1). Major exclusions and reasons for exclusion are detailed in Additional file 1: Table S1. These trials were published from 1984 to 2016. The median number of enrolled patients was 231 (IQR 90–430). Acute kidney injury was the most representative condition of interest (4 studies [18, 20, 22, 25]), followed by cardiac arrest (3 studies [11, 17, 21]) and sepsis (3 studies [14, 16, 19]). Standard care or conventional therapy was used as control in 7 studies [8, 9, 14, 16, 19, 21, 23]. The most common timing of significant mortality differences was hospital discharge (8 studies [9–11, 13, 17, 18, 21, 23]). The characteristics of the included sRCTs are described in Table 1. The vast majority of the sRCTs included in this study (18 out of 19) were assessed as having a low risk of bias, while the remaining trial was judged as having some concerns (see Additional file 1: Table S2).

**Table 1:** Single-center randomized trials with statistically significant survival benefits

References	Number of patients	Population	Intervention	Control	Mortality timepoint
Lachman et al. [14]	33	Septic shock	Anti-lipopolysaccharide immunoglobulin G	Conventional treatment	7.1% versus 47% Hospital discharge
Sack et al. [21]	103	In-hospital cardiac arrest	Interposed abdominal counterpulsation	Standard care	75% versus 93% Hospital discharge
Boyd et al. [8]	107	High-risk surgical patients	Supranormal oxygen delivery	Conventional therapy	5.7% versus 22% 28 days
Levacher et al. [15]	76	Upper gastrointestinal bleeding	Terlipressin plus glyceryl trinitrate	Placebo	20% versus 42% 15 days
Antonelli et al. [7]	40	Acute respiratory failure after solid organ transplantation	Non-invasive ventilation	Supplemental oxygen	20% versus 50% ICU discharge
Ronco et al. [20]	425	Acute renal failure in ICU	Higher intensity renal replacement therapy	Low or intermediate volume ultrafiltration	41% versus 57% versus 58% 15 days
Rivers et al. [19]	263	Sepsis and septic shock	Early goal-directed therapy	Standard care	44% versus 57% 60 days
Hilbert et al. [13]	52	Acute respiratory failure in immunosuppressed patients	Non-invasive ventilation	Supplemental oxygen	50% versus 81% Hospital discharge
van der Berghe et al. [23]	1548	ICU patients	Intensive insulin therapy	Conventional therapy	4.6% versus 8.0% Hospital discharge
Dorian et al. [11]	347	Out-of-hospital ventricular fibrillation	Amiodarone	Lidocaine	77% versus 88% Hospital discharge
Schiffl et al. [22]	160	Acute renal failure	Daily intermittent hemodialysis	Alternate-day hemodialysis	28% versus 46% 14 days
Phu et al. [18]	70	Acute renal failure with urgent renal replacement therapy indication	Venovenous dialysis	Peritoneal dialysis	15% versus 47% Hospital discharge
de Jonge et al. [9]	934	Patients on mechanical ventilation	Selective decontamination digestive tract	Standard care	24% versus 31% Hospital discharge
de Silva et al. [10]	401	Yellow-oleander poisoning	Charcoal	Placebo	2.5% versus 8.0% Hospital discharge
Olasveengen et al. [17]	1183	Out-of-hospital nontraumatic cardiac arrest	CPR with epinephrine administration	CPR without epinephrine administration	68% versus 79% Hospital admission
Morelli et al. [16]	154	Septic shock	Esmolol infusion	Standard care	49% versus 81% 28 days
Villanueva et al. [24]	921	Upper gastrointestinal bleeding	Restrictive transfusion strategy	Liberal transfusion strategy	5.0% versus 8.9% 45 days
Girardis et al. [12]	434	ICU patients	Conservative oxygen supplementation	Conventional oxygen supplementation	12% versus 20% ICU discharge
Zarbock et al. [25]	231	Acute kidney injury stage 2	Early renal replacement therapy	Delayed renal replacement therapy	39% versus 55% 90 days

*CPR* cardiopulmonary resuscitation, *ICU* intensive care unit

Most of the included sRCTs (16/19, 84%) [7, 9–17, 19, 20, 22–25] were followed by at least one subsequent mRCT (in total 24 studies [26–49]), while no mRCT was available for the remaining three studies [8, 18, 21]. The mRCTs enrolled more patients (median, 1192 [IQR 488–3021] vs. 231 [IQR 90–430] in sRCTs). The median number of participating centers was 29 (IQR 8–37) and one-third of the studies involved multiple countries [27, 29, 32, 34, 40, 41, 45, 48]. The median interval between the publications of a sRCT and its relevant subsequent mRCT was 8 years (IQR 5–13 years). Survival or mortality was the primary outcome in the 10 sRCTs and 17 mRCTs (as listed in Additional file 1: Table S3).

**Fig. 2:**
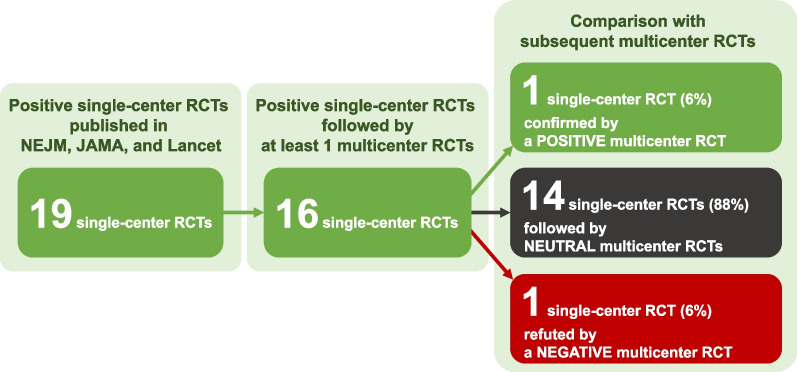
Mortality findings in multicenter randomized trials following positive single-center trials. *RCT* randomized controlled trials, *NEJM* New England Journal of Medicine

The survival benefits of one intervention (epinephrine during out-of-hospital cardiac arrest [17]) were confirmed by a subsequent mRCT [46]. Fourteen studies [9–13, 16, 19, 22, 24, 25] were followed by neutral mRCTs (no statistically significant mortality difference between groups) [26–33, 35–45, 47–49]. One sRCT reporting survival benefit of intensive insulin therapy [23] was contradicted by a large mRCT documenting a statistically significant mortality increase in patients randomized to the intensive insulin therapy arm [34]. Figure 2 and Table 2 describes the mortality findings of these mRCTs.

**Table 2:** Subsequent multicenter randomized trials and their mortality findings

sRCT	References of mRCT	Number of patients	Number of centers	Number of countries	Mortality
Anti-lipopolysaccharide immunotherapy in sepsis, 1984 [14]	Greenman et al. [37]	486	33	1	No statistically significant difference
	Angus et al. [26]	1102	136	1	No statistically significant difference
Interpose abdominal counterpulsation during cardiac arrest, 1992 [21]	Not available				
Supranormal oxygen delivery, 1993 [8]	Not available				
Terlipressin for upper gastrointestinal bleeding in cirrhotic patients, 1995 [15]	Feu et al. [33]	161	4	1	No statistically significant difference
	Escorsell et al. [32]	219	9	2	No statistically significant difference
Non-invasive ventilation in immunosuppressed patients, 2000 [7, 13]	Lemiale et al. [41]	374	28	2	No statistically significant difference
High-intensity renal replacement therapy in acute renal failure, 2000 [20]	Palevsky et al. [44]	1124	27	1	No statistically significant difference
	Bellomo et al. [29]	1508	35	2	No statistically significant difference
Early goal-directed therapy in sepsis, 2001 [19]	Peake et al. [45]	1600	51	5	No statistically significant difference
	Yealy et al. [49]	1341	31	1	No statistically significant difference
	Mouncey et al. [42]	1260	56	1	No statistically significant difference
Intensive insulin therapy, 2001 [23]	Finfer et al. [34]	6104	42	4	Statistically significant mortality increase at 90 days (28% vs. 25%)
Amiodarone in refractory ventricular fibrillation, 2002 [11]	Kudenchuk et al. [40]	3026	10^a^	2	No statistically significant difference
Daily hemodialysis in acute renal failure, 2002 [22]	Ponce et al. [47]	407	2	1	No statistically significant difference
Hemofiltration versus peritoneal dialysis in acute renal failure, 2002 [18]	Not available				
Selective decontamination of digestive tract, 2003 [9]	de Smet et al. [30]	5939	13	1	No statistically significant difference
	Myburgh et al. [43]	5982	19	1	No statistically significant difference
Charcoal in poisoning, 2003 [10]	Eddleston et al. [31]	4632	3	1	No statistically significant difference
Epinephrine during cardiopulmonary resuscitation, 2009 [17]	Perkins et al. [46]	8014	5^b^	1	Statistically significant mortality reduction at 30 days (3.2% vs 2.4%)
Beta blocker in sepsis, 2013 [16]	Kakihana et al. [39]	151	54	1	No statistically significant difference
Transfusion thresholds in upper gastrointestinal bleeding, 2013 [24]	Jairath et al. [38]	936	6	1	No statistically significant difference
Oxygen targets, 2016 [12]	Schjørring et al. [48]	2928	35	7	No statistically significant difference
	Gelissen et al. [36]	574	4	1	No statistically significant difference
Timing of renal replacement therapy in acute kidney injury, 2016 [25]	Gaudry et al. [35]	620	31	1	No statistically significant difference
	Barbar et al. [28]	488	29	1	No statistically significant difference
	Bagshaw et al. [27]	3019	168	15	No statistically significant difference

*CPR* cardiopulmonary resuscitation, *sRCT* single-center randomized controlled trial, *mRCT* multicenter randomized controlled trial

^a^Number of clinical centers consisting of the Resuscitation Outcomes Consortium

^b^Number of National Health Service ambulance services

**Fig. 3:**
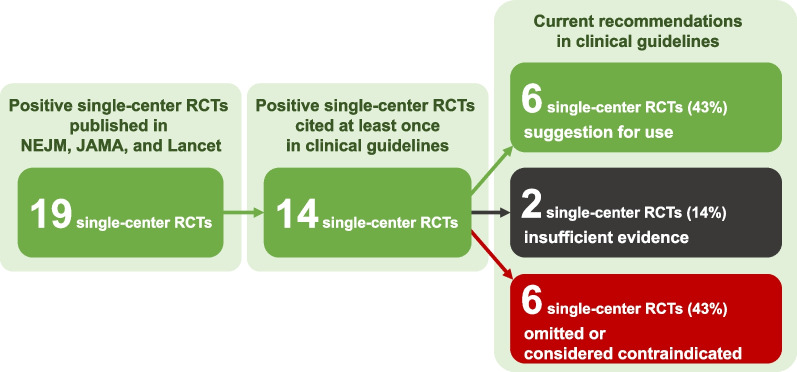
Current guideline recommendations of positive single-center trials

Figure 3 and Table 3 summarizes how clinical guidelines have considered survival benefits shown in sRCTs. Among the included 19 sRCTs, 14 were cited in clinical guidelines at least once (13 sRCTs with subsequent mRCTs and one without) [7, 9, 11–13, 15, 17, 19–25]. Among the 13 sRCTs followed by mRCTs, the guidelines initially provided recommendations or suggestions based on the positive results of seven sRCTs [7, 11, 13, 15, 19, 23, 24]. However, the current guidelines do not support applying two of these interventions anymore [19, 23]. Treatments assessed in the remaining five sRCTs [7, 11, 13, 15, 24] remained as suggestions for use in clinical guidelines. Such suggestions remained even after the publication of mRCTs, which reported neutral mortality findings.

**Table 3:** Citation of single-center randomized trials in international clinical guidelines

Research topic	The first guideline citating the sRCT	Recommendation in the first guideline citing the sRCT	Guideline removing or suggesting against the sRCT	Current recommendation in guidelines
*16 sRCTs with subsequent mRCTs*				
Anti-lipopolysaccharide immunotherapy in sepsis, 1984 [14]	Not available	Not applicable	Not applicable	Not applicable
Terlipressin for upper gastrointestinal bleeding in cirrhotic patients, 1995 [15]	2018 [50]	Suggested	Not applicable	Suggested [50]
Non-invasive ventilation in immunosuppressed patients, 2000 [7]	2015 [51]	Some evidence of benefit in certain patients with immunosuppression	2020 [52]	Suggested [52]
High-volume ultrafiltration in acute renal failure, 2000 [20]	2008 [53]	Insufficient evidence	2021 [54]	Insufficient evidence [54]
Non-invasive ventilation in immunosuppressed patients, 2001 [13]	2015 [51]	Some evidence of benefit in certain patients with immunosuppression	2023 [55]	Suggested [55]
Early goal-directed therapy in sepsis, 2001 [19]	2004 [56]	Recommended	2017 [57]	Not mentioned [54]
Intensive insulin therapy, 2001 [23]	2004 [56]	Recommended with a glucose goal of < 150 mg/dL	2013 [58]	Suggested against [54]
Amiodarone in refractory ventricular fibrillation, 2002 [11]	2006 [59]	Recommended	2019 [60]	Suggested [61]
Daily hemodialysis in acute renal failure, 2002 [22]	2008 [53]	Insufficient evidence	2013 [58]	Not mentioned [54]
Selective decontamination of digestive tract, 2003 [9]	2008 [53]	No recommendation	2016 [57]	Not mentioned [54]
Charcoal in poisoning, 2003 [10]	Not available	Not applicable	Not applicable	Not applicable
Epinephrine during cardiopulmonary resuscitation, 2009 [17]	2010 [62]	Insufficient evidence	2015 [63]	Recommended [64]
Beta blocker in sepsis, 2013 [16]	Not available	Not applicable	Not applicable	Not applicable
Restrictive transfusion threshold in upper gastrointestinal bleeding, 2013 [24]	2016 [65]	Recommended	Still cited in the current guideline [66]	Suggested [66]
Oxygen targets, 2016 [12]	2021 [54]	Insufficient evidence	Not available	Insufficient evidence [54]
Early initiation of renal replacement therapy in acute kidney injury, 2016 [25]	2021 [54]	Suggested against	Not available	Suggested against [54]
*3 sRCTs without subsequent mRCTs*				
Interpose abdominal counterpulsation during cardiac arrest, 1992 [21]	2005 [67]	No clear statement	2015 [63]	Not mentioned [64]
Supranormal oxygen delivery, 1993 [8]	Not available	Not applicable	Not available	Not applicable
Hemofiltration vs peritoneal dialysis in acute renal failure, 2002 [18]	Not available	Not applicable	Not applicable	Not applicable

*CPR* cardiopulmonary resuscitation, *sRCT* single-center randomized controlled trial

Of the remaining six sRCTs for which guidelines did not support the intervention investigated, five [9, 12, 17, 20, 22] were cited in the guidelines without a clear recommendation, primarily due to inadequate evidence. However, one intervention—epinephrine for cardiac arrest—that exhibited survival benefits in the sRCT [17] and subsequent mRCT [46] is currently recommended in guidelines. The remaining sRCT [25] was not endorsed by the initial relevant guideline as a result of other mRCTs that showed no significant mortality reduction.

Finally, among three sRCTs which has not had a subsequent mRCT, only one study [21] was cited in guidelines without any recommendation but is not referenced in the current guidelines.

Consequently, among 14 sRCTs originally referenced to in clinical guidelines, six (43%) are still cited to suggest for the intervention in current international guidelines [7, 11, 13, 15, 17, 24]. Conversely, six other sRCTs (43%) were either omitted or considered contraindicated in subsequent guideline versions [9, 19, 22, 23, 25]. Among these six studies, the median duration from the initial citation in the guidelines to an alteration in recommendation against its use or removal from guidelines was 9 years (IQR 6–12 years). Regarding the remaining two studies [12, 20], no recommendation was made due to insufficient evidence.

A sensitivity analysis restricted to recent sRCTs confirmed the overall results: survival benefits were infrequently replicated in subsequent mRCTs; half of the positive sRCTs were omitted or considered contraindicated in the current guidelines (detailed in Table S4 in the Additional file 1).

## Discussion

### Key findings

Our systematic review found 19 sRCTs with a statistically significant mortality decrease in critically ill adult patients. Most of these were followed by at least one subsequent mRCT. Survival benefits observed in sRCTs were rarely corroborated by mRCTs, with most mRCTs reporting neutral results on mortality, and one mRCT finding a significant mortality increase with intensive glucose control. Treatment recommendations based on the initial citation of sRCTs with survival benefits were included in international guidelines and typically remained unchanged for a decade before any revisions were made based on subsequent relevant mRCTs.

### Relationship with previous literature

RCTs in intensive care medicine tend to deliver neutral results in terms of mortality for several reasons including heterogeneity of patient characteristics, underlying practice variation, insufficient power, and likely small treatment effects [3]. This fact poses an important challenge for clinicians because they must perform clinical practice without robust evidence supporting their decisions. As a result, positive trials, namely RCTs reporting statistically significant reductions in mortality attributable to the intervention of interest, look attractive and are often taken up by physicians to change their routine management. Unfortunately, however, such positive RCTs frequently suffer from methodological problems, which can limit the applicability of their findings. Furthermore, single-centric design itself carries many other limitations [2, 3].

One of the major challenges of sRCTs is that, to achieve an effect on mortality in the presence of a small sample size, they must achieve an implausibly large effect size. Single-center trials are typically conducted by advocates of the intervention under investigation [2]. The delivery of such interventions generally requires specialized expertise and dedication, which may not be readily transferable to other centers involved in subsequent large mRCTs. Such discrepancies may limit the feasibility of the interventions, potentially diminishing the magnitude of the treatment effects observed in mRCTs compared to sRCTs. In fact, a meta-epidemiological study evaluated the differences in treatment effects between sRCTs and multicenter RCTs and found that single-center trials showed a statistically significant larger treatment effects than multicenter trials (ratio of odds ratios, 0.73; 95% confidence interval 0.64–0.83) [68]. This finding was confirmed by a systematic review assessing treatment effects on mortality in critically ill settings [69]. By pooling 82 eligible RCTs, this systematic review found that a single-center design resulted in larger treatment effects than a multicenter design (ratio of odds ratios, 0.64; 95% confidence interval 0.47–0.87) [69]. Our selection criterion of sRCTs with significant mortality differences is a unique approach; nonetheless, the present systematic review was consistent with previous work showing that survival benefits in sRCTs were rarely replicated in mRCTs. Moreover, we identified one mRCT demonstrating a significant mortality increase by an intervention (intensive glucose control strategy) [34], which reduced mortality within the context of a previous single-center trial [23].

Nearly half of clinical guidelines that cite sRCTs, recommend the relevant intervention based on their positive results, despite some of these endorsements being subsequently refuted in light of accumulated evidence. In addition, a decade was typically required to amend such recommendations from clinical guidelines. Given the pervasive application of interventions examined in RCTs, these initial recommendations might have played a substantial role in the potential consequences on patient outcomes, healthcare resources, and economic costs. For example, early-goal directed therapy was recommended in the surviving sepsis campaign guidelines in 2004 [56]. However, later, three mRCTs found no benefits in clinically relevant outcomes [42, 45, 49]. Furthermore, economic evaluation using one of the mRCTs revealed that early-goal directed therapy was associated with increased health care costs without improving outcomes [70].

Despite these methodological challenges, positive sRCTs have made changes in clinical practice. The early goal-directed therapy for septic shock is a typical example. The initial sRCT [19] showed survival benefits of this protocolized management, which was not replicated in subsequent mRCTs [42, 45, 49]. However, given the difference in patient severity between the sRCT and subsequent mRCTs (e.g., reduced vs. normal central venous oxygen saturation [ScvO_2_]), clinicians now pay more attention to ScvO_2_ values than before the sRCT [71]. Furthermore, the lack of multicentric confirmation of survival benefits implies a restricted external validity of sRCTs rather than an indication of them producing false positive results.

As the intensive care community advances the methodology of randomized trial design and execution, there remains a notable lack of evidence demonstrating improved mortality from interventions. These disappointing results have been obtained by using frequentist statistics, where the conclusion is dichotomized to yes or no based on confidence intervals and *p* values. In contrast, Bayesian analysis provides a probabilistic assessment of the magnitude and direction of true treatment effects, which allows clinicians to augment the interpretation of the trial results. Interestingly, there are several intensive care trials where frequentist statistics denied significant mortality reduction, followed by a Bayesian reanalysis revealing a high probability of survival benefits [72, 73]. Therefore, the integration of Bayesian analysis in intensive care trials may offer a solution to the limitations commonly encountered with frequentist approaches.

### Implications

This systematic review found mortality reduction was rarely replicated in mRCTs despite the existence of previous positive sRCTs. This implies that there are potential risks when incorporating novel interventions into routine practice based on positive sRCTs without mRCTs confirmation. Importantly, no intervention is free from complications. In addition, new interventions tested in randomized trials often consume more human resources and economic costs. Therefore, management change will inevitably result in complications, increased workload, and costs, all of which did not exist with previous usual care. Given the high likelihood of no mortality difference or even mortality increase in subsequent mRCTs, our findings imply that clinicians should wait for a large-scale trial prior to changing practice or at least be very careful in interpreting the results of positive sRCTs.

### Strengths and limitations

This systematic review is the first study to comprehensively identify sRCTs reporting statistically significant reductions in mortality and their corresponding subsequent mRCTs in the field of intensive care medicine. The infrequent replicability of survival benefits in mRCTs corroborated the limited generalizability of sRCTs’ findings. Evaluating the impact of sRCTs on clinical guidelines may be a novel approach, but it yields important insights into the development and interpretation of guidelines.

We acknowledge several limitations. First, given our focus was solely on intensive care sRCTs, our findings may not translate to other medical disciplines. Nevertheless, the generic limitations of sRCTs are universal, regardless of the targeted population or intervention type. As such, sRCTs need to be perceived as hypothesis-generating and clinicians ought to assess the results of sRCTs with a balanced consideration of their strengths and weaknesses. Second, our study included only sRCTs published until 2016, thereby excluding more recent sRCTs. Despite this limitation, our primary objective was to compare the mortality findings of sRCTs with those of subsequent mRCTs, which necessitated an intervening period between them. In addition, the median duration between sRCT and mRCT publication was 8 years, providing justification for our inclusion criteria. Third, we included positive sRCTs published in the three renowned general medical journals, excluding those in intensive care specialty journals (as listed in Table S5: Additional file 1). As a result, the number of eligible studies was relatively small; nonetheless, we employed this strategy to evaluate whether subsequent mRCTs could replicate the survival benefits observed in sRCTs with rigorous methodologies. Given the high standards of the included studies and the concordance of the results with previous literature, it is plausible that our findings could be extrapolated to positive sRCTs reported in specialty journals. Finally, our search was confined to international guidelines to explore sRCTs’ citations. This approach was chosen to ensure the quality of evidence synthesis and generalized perspective.

## Conclusions

Our systematic review found that the statistically significant survival improvement shown in sRCTs was rarely confirmed by multicenter randomized evidence in intensive care settings. Clinicians should be cautious in altering routine clinical practices until well-conducted multicenter randomized trials are available. Given their substantial implications for global clinical practice, international guidelines should refrain from issuing a clear recommendation based solely on the positive results of sRCTs.

### Supplementary Information

**Additional file 1. **Search strategy, PRISMA checklist, Supplementary tables, and Supplementary references.

## Data Availability

We collected the summary data from published randomized trials. This published article and its supplementary files include all the data generated or analyzed for this study. Further information is available from the corresponding authors upon reasonable request.

## Abbreviations

ICU: Intensive care unit
IQR: Interquartile range
mRCT: Multicenter randomized controlled trial
PRISMA: Preferred reporting items for systematic reviews and meta-analyses
sRCT: Single-center randomized controlled trial
RCT: Randomized controlled trial
