# Supplementary material for: Positive single-center randomized trials and subsequent multicenter randomized trials in critically ill patients: a systematic review
Source: Crit Care. 2023 Nov 28;27:465. doi: 10.1186/s13054-023-04755-5 (PMC10685543; doi:10.1186/s13054-023-04755-5)
Supplement: Supplementary file 1 — Additional file 1. Search strategy, PRISMA checklist, Supplementary tables, and Supplementary references. [file 13054_2023_4755_MOESM1_ESM.docx]

**Additional file 1**

**Positive single-center randomized trials and subsequent multicenter randomized trials in critically ill patients: a systematic review**

**Authors**

Yuki Kotani, Stefano Turi, Alessandro Ortalda, Martina Baiardo Redaelli, Cristiano Marchetti, Giovanni Landoni, Rinaldo Bellomo

**Table of contents**

Search strategy for systematic literature review 3

PRISMA 2020 checklist. 4

Table S1. Major exclusions and reasons for exclusion, in order of year of publication. 7

Table S2. Risk of bias assessment of included studies. 9

Table S3. Primary endpoint in the single-center and subsequent multicenter randomized trials. 10

Table S4. Sensitivity analysis in recent positive single-center randomized trials 12

Table S5. Single-center randomized trials reporting significant survival benefits and published in six intensive care specialty journals. 13

Supplementary references 15

# Search strategy for systematic literature review

PubMed

((dead[tiab] or death[tiab] or die[tiab] or died[tiab] or mortality[tiab] or fatalit*[tiab] or exitus[tiab] or surviv*[tiab]) and ("anesthesia"[tiab] OR "cardiac arrest"[tiab] or "critical care"[tiab] or sepsis[tiab] or "critical illness"[tiab] or "critically ill" [tiab] or "ARDS"[TIAB] or "acute respiratory distress syndrome"[tiab] OR "ecmo"[tiab] OR "intensive care"[tiab] or emergen[tiab]) AND ((randomized controlled trial[pt] OR controlled clinical trial[pt] OR randomized controlled trials[mh] OR random allocation[mh] OR double-blind method[mh] OR single-blind method[mh] OR clinical trial[pt] OR clinical trials[mh] OR (clinical trial[tw] OR ((singl*[tw] OR doubl*[tw] OR trebl*[tw] OR tripl*[tw]) AND (mask*[tw] OR blind[tw])) OR (latin square[tw]) OR placebos[mh] OR placebo*[tw] OR random*[tw] OR research design[mh:noexp] OR comparative study[tw] OR follow-up studies[mh] OR prospective studies[mh] OR cross-over studies[mh] OR control*[tw] OR prospectiv*[tw] OR volunteer*[tw]) NOT (animal[mh] NOT human[mh]))) AND (:"1900/01/01"[PDAT] : "2016/12/31"[PDAT])

# PRISMA 2020 checklist.

| **Section and Topic** | **Item #** | **Checklist item** | **Location where item is reported** |
| --- | --- | --- | --- |
| **TITLE** | | |  |
| Title | 1 | Identify the report as a systematic review. | 1 |
| **ABSTRACT** | | |  |
| Abstract | 2 | See the PRISMA 2020 for Abstracts checklist. | 2 |
| **INTRODUCTION** | | |  |
| Rationale | 3 | Describe the rationale for the review in the context of existing knowledge. | 4 |
| Objectives | 4 | Provide an explicit statement of the objective(s) or question(s) the review addresses. | 4 |
| **METHODS** | | |  |
| Eligibility criteria | 5 | Specify the inclusion and exclusion criteria for the review and how studies were grouped for the syntheses. | 5 |
| Information sources | 6 | Specify all databases, registers, websites, organisations, reference lists and other sources searched or consulted to identify studies. Specify the date when each source was last searched or consulted. | 5 |
| Search strategy | 7 | Present the full search strategies for all databases, registers and websites, including any filters and limits used. | 5, Additional file 1 |
| Selection process | 8 | Specify the methods used to decide whether a study met the inclusion criteria of the review, including how many reviewers screened each record and each report retrieved, whether they worked independently, and if applicable, details of automation tools used in the process. | 5 |
| Data collection process | 9 | Specify the methods used to collect data from reports, including how many reviewers collected data from each report, whether they worked independently, any processes for obtaining or confirming data from study investigators, and if applicable, details of automation tools used in the process. | 5–6 |
| Data items | 10a | List and define all outcomes for which data were sought. Specify whether all results that were compatible with each outcome domain in each study were sought (e.g. for all measures, time points, analyses), and if not, the methods used to decide which results to collect. | 5 |
|  | 10b | List and define all other variables for which data were sought (e.g. participant and intervention characteristics, funding sources). Describe any assumptions made about any missing or unclear information. | 5 |
| Study risk of bias assessment | 11 | Specify the methods used to assess risk of bias in the included studies, including details of the tool(s) used, how many reviewers assessed each study and whether they worked independently, and if applicable, details of automation tools used in the process. | 5 |
| Effect measures | 12 | Specify for each outcome the effect measure(s) (e.g. risk ratio, mean difference) used in the synthesis or presentation of results. | 6 |
| Synthesis methods | 13a | Describe the processes used to decide which studies were eligible for each synthesis (e.g. tabulating the study intervention characteristics and comparing against the planned groups for each synthesis (item #5)). | 6 |
|  | 13b | Describe any methods required to prepare the data for presentation or synthesis, such as handling of missing summary statistics, or data conversions. | 6 |
|  | 13c | Describe any methods used to tabulate or visually display results of individual studies and syntheses. | 6 |
|  | 13d | Describe any methods used to synthesize results and provide a rationale for the choice(s). If meta-analysis was performed, describe the model(s), method(s) to identify the presence and extent of statistical heterogeneity, and software package(s) used. | 6 |
|  | 13e | Describe any methods used to explore possible causes of heterogeneity among study results (e.g. subgroup analysis, meta-regression). | 6 |
|  | 13f | Describe any sensitivity analyses conducted to assess robustness of the synthesized results. | 6 |
| Reporting bias assessment | 14 | Describe any methods used to assess risk of bias due to missing results in a synthesis (arising from reporting biases). | 5 |
| Certainty assessment | 15 | Describe any methods used to assess certainty (or confidence) in the body of evidence for an outcome. | NA |
| **RESULTS** | | |  |
| Study selection | 16a | Describe the results of the search and selection process, from the number of records identified in the search to the number of studies included in the review, ideally using a flow diagram. | 6 |
|  | 16b | Cite studies that might appear to meet the inclusion criteria, but which were excluded, and explain why they were excluded. | 6 |
| Study characteristics | 17 | Cite each included study and present its characteristics. | 6, Table 1 |
| Risk of bias in studies | 18 | Present assessments of risk of bias for each included study. | 6, Supplemental Table 2 |
| Results of individual studies | 19 | For all outcomes, present, for each study: (a) summary statistics for each group (where appropriate) and (b) an effect estimate and its precision (e.g. confidence/credible interval), ideally using structured tables or plots. | 6–7 |
| Results of syntheses | 20a | For each synthesis, briefly summarise the characteristics and risk of bias among contributing studies. | 6–7 |
|  | 20b | Present results of all statistical syntheses conducted. If meta-analysis was done, present for each the summary estimate and its precision (e.g. confidence/credible interval) and measures of statistical heterogeneity. If comparing groups, describe the direction of the effect. | 6–7 |
|  | 20c | Present results of all investigations of possible causes of heterogeneity among study results. | 6–7 |
|  | 20d | Present results of all sensitivity analyses conducted to assess the robustness of the synthesized results. | 6–7 |
| Reporting biases | 21 | Present assessments of risk of bias due to missing results (arising from reporting biases) for each synthesis assessed. | NA |
| Certainty of evidence | 22 | Present assessments of certainty (or confidence) in the body of evidence for each outcome assessed. | NA |
| **DISCUSSION** | | |  |
| Discussion | 23a | Provide a general interpretation of the results in the context of other evidence. | 8–9 |
|  | 23b | Discuss any limitations of the evidence included in the review. | 10 |
|  | 23c | Discuss any limitations of the review processes used. | 10 |
|  | 23d | Discuss implications of the results for practice, policy, and future research. | 9–10 |
| **OTHER INFORMATION** | | |  |
| Registration and protocol | 24a | Provide registration information for the review, including register name and registration number, or state that the review was not registered. | 4 |
|  | 24b | Indicate where the review protocol can be accessed, or state that a protocol was not prepared. | 4 |
|  | 24c | Describe and explain any amendments to information provided at registration or in the protocol. | NA |
| Support | 25 | Describe sources of financial or non-financial support for the review, and the role of the funders or sponsors in the review. | 12 |
| Competing interests | 26 | Declare any competing interests of review authors. | 12 |
| Availability of data, code and other materials | 27 | Report which of the following are publicly available and where they can be found: template data collection forms; data extracted from included studies; data used for all analyses; analytic code; any other materials used in the review. | 12 |

#

# Table S1. Major exclusions and reasons for exclusion, in order of year of publication.

| **First author, year** | **Reason for exclusion** |
| --- | --- |
| Ziegler EJ, 1982 [1] | Multicentric design |
| Barer D, 1983 [2] | Multicentric design |
| Bone RC, 1987 [3] | Multicentric design |
| Greenman RL, 1991 [4] | Multicentric design |
| Gutierrez G, 1992 [5] | Multicentric design |
| Cohen TJ, 1993 [6] | A significant mortality difference ≤ 48 hours |
| Hayes MA, 1994 [7] | Multicentric design |
| Abraham E, 1995 [8] | Multicentric design |
| Brochard L, 1995 [9] | Multicentric design |
| Sainio V, 1995 [10] | Non-critically ill patients |
| Mangano DT, 1996 [11] | Non-critically ill patients |
| Lindner KH, 1997 [12] | A significant mortality difference at ≤ 48 hours |
| Meduri G, 1998 [13] | Multicentric design |
| Amato MB, 1998 [14] | Multicentric design |
| Gueugniaud PY, 1998 [15] | Multicentric design |
| Kudenchuk J, 1999 [16] | Multicentric design |
| Takala J, 1999 [17] | Multicentric design |
| Sort P, 1999 [18] | Multicentric design |
| Sloan EP, 1999 [19] | Multicentric design |
| Poldermans D, 1999 [20] | Multicentric design |
| Plant PK, 2000 [21] | Multicentric design |
| Brower RG, 2000 [22] | Multicentric design |
| Nouira S, 2001 [23] | Multicentric design |
| Warren BL, 2001 [24] | Multicentric design |
| Bernard GR, 2001 [25] | Multicentric design |
| Gattinoni L, 2001 [26] | Multicentric design |
| O’Grady JG, 2002 [27] | Multicentric design |
| Hypothermia after Cardiac Arrest Study Group, 2002 [28] | Multicentric design |
| Annane D, 2002 [29] | Multicentric design |
| Corwin HL, 2002 [30] | Multicentric design |
| Marenzi G, 2003 [31] | Non-critically ill patients |
| Esteban A, 2004 [32] | Multicentric design |
| Roberts I, 2004 [33] | Multicentric design |
| Hallstrom AP, 2004 [34] | Multicentric design |
| Chen ZM, 2005 [35] | Multicentric design |
| Steinberg KP, 2006 [36] | Multicentric design |
| Hallstrom A, 2006 [37] | Multicentric design |
| Corwin HL, 2007 [38] | Multicentric design |
| SAFE Study Investigators, 2007 [39] | Multicentric design |
| POISE Study Group, 2008 [40] | Multicentric design |
| Fergusson DA, 2008 [41] | Multicentric design |
| Girard TD, 2008 [42] | Multicentric design |
| Russell JA, 2008 [43] | Multicentric design |
| Cruz DN, 2009 [44] | Multicentric design |
| NICE-SUGAR Study Investigators, 2009 [45] | Multicentric design |
| Ferrer M, 2009 [46] | Multicentric design |
| CRASH-2 trial collaborators, 2010 [47] | Multicentric design |
| De Backer D, 2010 [48] | Multicentric design |
| Papazian L, 2010 [49] | Multicentric design |
| Aufderheide TP, 2011 [50] | Multicentric design |
| Gao Smith F, 2012 [51] | Multicentric design |
| Perner A, 2012 [52] | Multicentric design |
| Thielmann M, 2013 [53] | Non-critically ill patients |
| Annane D, 2013 [54] | Multicentric design |
| Mentzelopoulos SD, 2013 [55] | Multicentric design |
| Guérin C, 2013 [56] | Multicentric design |
| Mourvillier B, 2013 [57] | Multicentric design |
| Ferguson ND, 2013 [58] | Multicentric design |
| Villanueva C, 2013 | Multicentric design |
| Van Zanten ARH, 2014 [59] | Multicentric design |
| Amrein K, 2014 [60] | No significant mortality difference |
| Oostdijk EAN, 2014 [61] | Multicentric design |
| Caironi P, 2014 [62] | Multicentric design |
| Frat JP, 2015 [63] | Multicentric design |
| Murphy GJ, 2015 [64] | Multicentric design |
| Andrews PJ, 2015 [65] | Multicentric design |

#

# Table S2. Risk of bias assessment of included studies.


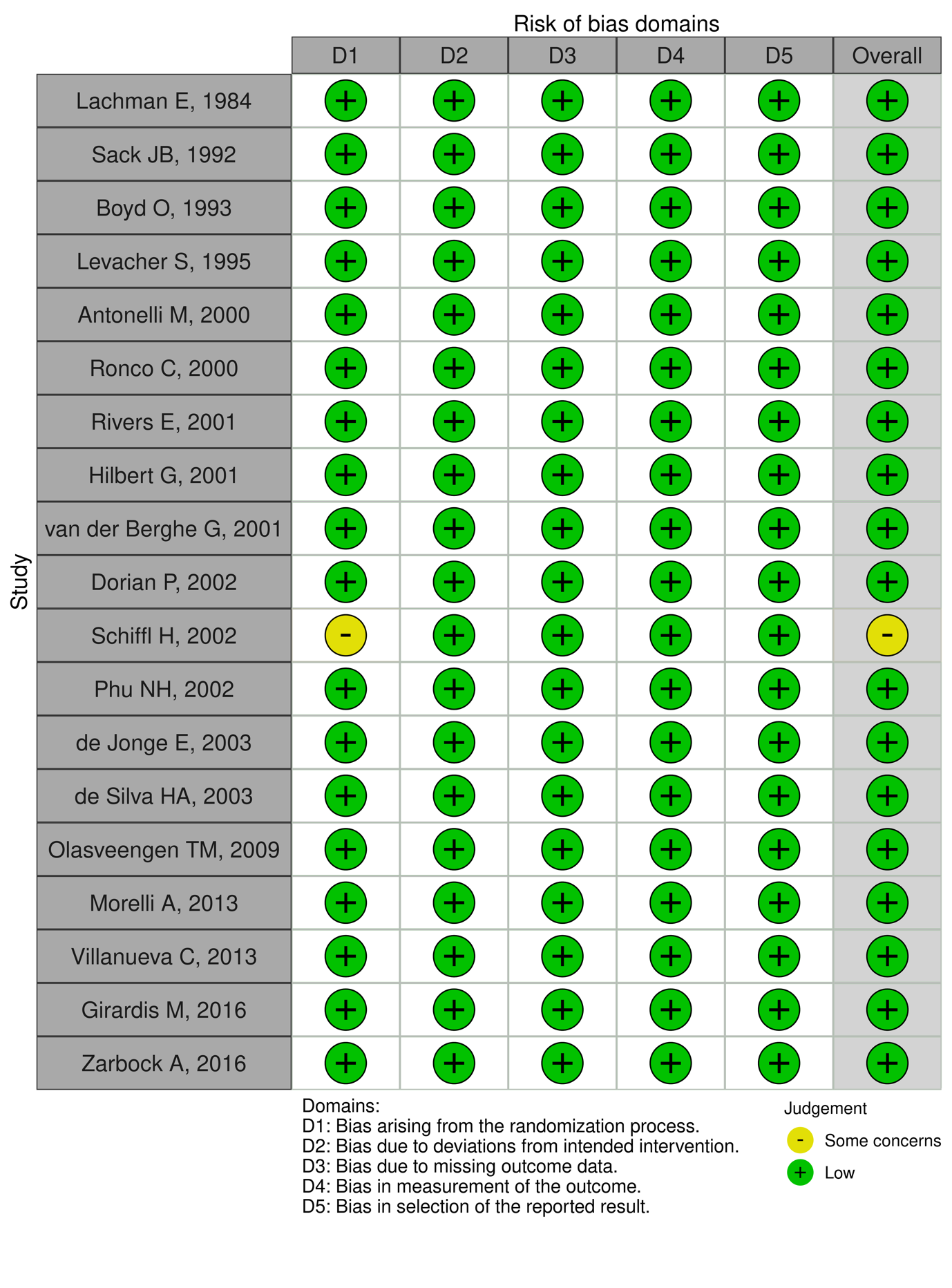


# Table S3. Primary endpoint in the single-center and subsequent multicenter randomized trials.

| Research topic | Single-center RCT | Primary endpoint | Multicenter RCT | Primary endpoint |
| --- | --- | --- | --- | --- |
| Anti-lipopolysaccharide immunotherapy in sepsis | Lachman E, 1984 [66] | Hospital mortality | Greenman RL, 1991 [4] | 30-day mortality |
| Terlipressin for upper gastrointestinal bleeding in cirrhotic patients | Levacher S, 1995 [67] | Bleeding control | Feu F, 1996 [68] | Bleeding control |
| Non-invasive ventilation in immunosuppressed patients | Antonelli M, 2000 [69] | Need for intubation | Lemiale V, 2015 [70] | 28-day mortality |
|  | Hilbert G, 2001 [71] | Need for intubation |  |  |
| High-intensity renal replacement therapy in acute renal failure | Ronco C, 2000 [72] | Survival at 15 days after stopping hemofiltration | Palevsky PM, 2008 [73] | 60-day mortality |
|  |  |  | Bellomo R, 2009 [74] | 90-day mortality |
| Early goal-directed therapy in sepsis | Rivers E, 2001 [75] | Hospital mortality | Peake SL, 2014 [76] | 90-day mortality |
|  |  |  | Yealy DM, 2014 [77] | 60-day mortality |
|  |  |  | Mouncey PR, 2015 [78] | 90-day mortality |
| Intensive insulin therapy | van der Berghe G, 2001 [79] | Intensive care mortality | Finfer S, 2009 [80] | 90-day mortality |
| Amiodarone in refractory ventricular fibrillation | Dorian P, 2002 [81] | Survival to hospital admission | Kudenchuk PJ, 2016 [82] | Survival to hospital discharge |
| Selective digestive decontamination | de Jonge E, 2003 [83] | Hospital mortality | de Smet AM, 2009 [84] | 28-day mortality |
|  |  |  | Myburgh JA, 2022 [85] | 90-day mortality |
| Charcoal in poisoning | de Silva HA, 2003 [86] | Hospital mortality | Eddleston M, 2008 [87] | Hospital mortality |
| Epinephrine during cardiopulmonary resuscitation | Olasveengen TM, 2009 [88] | Survival to hospital discharge | Perkins GD, 2018 [89] | 30-day survival |
| Beta blocker in sepsis | Morelli A, 2013 [90] | Reduction in heart rate | Kakihana Y, 2020 [91] | Heart rate control at 24 hours |
| Transfusion thresholds in upper gastrointestinal bleeding | Villanueva C, 2013 [92] | 45-day mortality | Jairath V, 2015 [93] | Protocol adherence |
| Oxygen targets | Girardis M, 2016 [94] | Intensive care unit mortality | Schjørring OL, 2021 [95] | 90-day mortality |
|  |  |  | Gelissen H, 2021 [96] | Non-respiratory components of the Sequential Organ Failure Assessment score |
| Timing of renal replacement therapy | Zarbock A, 2016 [97] | 90-day mortality | Gaudry S, 2016 [98] | 60-day survival |
|  |  |  | Barbar SD, 2018 [99] | 90-day mortality |
|  |  |  | Bagshaw SM, 2020 [100] | 90-day mortality |

RCT: randomized controlled trial

# Table S4. Sensitivity analysis in recent positive single-center randomized trials

| **Mortality results in subsequent multicenter randomized trials** | **N =12** |
| --- | --- |
| Positive (significant mortality reduction) | 1 (8.3%) |
| Neutral (no significant mortality difference) | 10 (83%) |
| Negative (significant mortality increase) | 1 (8.3%) |
| **Guideline recommendations** | **N=10** |
| Suggestion for use | 4 (40%) |
| Inadequate evidence | 1 (10%) |
| Removal or Contraindication | 5 (50%) |

# Table S5. Single-center randomized trials reporting significant survival benefits and published in the six highest impact factor intensive care specialty journals.

| **Author, year** | **Journal** | **Number of patients** | **Population** | **Intervention** | **Control** | **Mortality (%) and timepoints** |
| --- | --- | --- | --- | --- | --- | --- |
| Shoemaker WC, 1988 [101] | Chest | 276 | High-risk surgery | Supranormal hemodynamic goals | Normal hemodynamic goals | 19% vs 34%  15 days |
| Ulrich C, 1989 [102] | ICM | 100 | ICU patients | Selective decontamination of the digestive tract | Standard care | 31% vs 54%  Not specified |
| Rocha LA, 1992 [103] | ICM | 101 | ICU patients | Selective decontamination of the digestive tract | Placebo | 21% vs 44%  Hospital discharge |
| DeRiso AJ II, 1996 [104] | Chest | 353 | Cardiac surgery | Chlorhexidine oral rinse | Placebo | 1.16% vs 5.56%  Hospital discharge |
| Yu M, 1998 [105] | CCM | 105 | Critically ill surgical patients | High oxygen delivery goal | Normal oxygen delivery goal | 33% vs 56%  14 days |
| Lobo SM, 2000 [106] | CCM | 37 | Moderate or high-risk surgery | Supranormal hemodynamic goals | Normal hemodynamic goals | 15.7% vs 50%  60 days |
| Auriant I, 2001 [107] | AJRCCM | 48 | Acute respiratory failure after lung resection | Noninvasive ventilation | Oxygen supplementation | 12.5% vs 37.5%  120 days |
| Goeters C, 2002 [108] | CCM | 144 | ICU patients | L-alanyl-L-glutamine | Standard parenteral nutrition | 33.3% vs 60%  6 months |
| Jacobs S, 2003 [109] | CCM | 71 | Septic shock | Fluconazole | Placebo | 22% vs 54%  30 days |
| Garrel D, 2003 [110] | CCM | 41 | Burn | Glutamine | Isonitrogenous control mixture | 11% vs 55%  Not specified |
| Pontes-Arruda A, 2006 [111] | CCM | 165 | Severe sepsis and septic shock | Diet enriched with EPA, GLA, and elevated antioxidants | Isonitrogenous and isocaloric control diet | 32.7% vs 52.1%  28 days |
| Fuhrmann JT, 2008 [112] | CCM | 32 | Refractory cardiogenic shock | Levosimendan | Enoximone | 31% vs 63%  30 days |
| Xiao G, 2008 [113] | CC | 159 | Acute severe traumatic brain injury | Progesterone | Placebo | 18% vs 32%  6 months |
| Ornico SR, 2013 [114] | CC | 40 | Extubated ICU patients | Noninvasive ventilation | Oxygen supplementation | 0% vs 22.2%  Hospital discharge |
| Vahedian-Azimi A, 2016 [115] | CC | 80 | In-hospital cardiac arrest | CPR with feedback device | Conventional CPR | 28% vs 65%  Return of spontaneous circulation |

Six critical care specialty journals are Lancet Respiratory Medicine, Intensive Care Medicine (ICM), American Journal of Respiratory and Critical Care Medicine (AJRCCM), Critical Care (CC), Chest, and Critical Care Medicine (CCM).

Abbreviations: CPR, cardiopulmonary resuscitation; EPA, eicosapentaenoic acid; GLA, gamma-linolenic acid; ICU, intensive care unit.

# Supplementary references

1. Ziegler EJ, McCutchan JA, Fierer J, Glauser MP, Sadoff JC, Douglas H, et al. Treatment of gram-negative bacteremia and shock with human antiserum to a mutant Escherichia coli. N Engl J Med. 1982;307:1225–30.

2. Barer D, Ogilvie A, Henry D, Dronfield M, Coggon D, French S, et al. Cimetidine and tranexamic acid in the treatment of acute upper-gastrointestinal-tract bleeding. N Engl J Med. 1983;308:1571–5.

3. Bone RC, Fisher CJ Jr, Clemmer TP, Slotman GJ, Metz CA, Balk RA. A controlled clinical trial of high-dose methylprednisolone in the treatment of severe sepsis and septic shock. N Engl J Med. 1987;317:653–8.

4. Greenman RL, Schein RM, Martin MA, Wenzel RP, MacIntyre NR, Emmanuel G, et al. A controlled clinical trial of E5 murine monoclonal IgM antibody to endotoxin in the treatment of gram-negative sepsis. The XOMA Sepsis Study Group. JAMA. 1991;266:1097–102.

5. Gutierrez G, Palizas F, Doglio G, Wainsztein N, Gallesio A, Pacin J, et al. Gastric intramucosal pH as a therapeutic index of tissue oxygenation in critically ill patients. Lancet. 1992;339:195–9.

6. Cohen TJ, Goldner BG, Maccaro PC, Ardito AP, Trazzera S, Cohen MB, et al. A comparison of active compression-decompression cardiopulmonary resuscitation with standard cardiopulmonary resuscitation for cardiac arrests occurring in the hospital. N Engl J Med. 1993;329:1918–21.

7. Hayes MA, Timmins AC, Yau E, Palazzo M, Hinds CJ, Watson D. Elevation of Systemic Oxygen Delivery in the Treatment of Critically Ill Patients. N Engl J Med. 1994;330:1717–22.

8. Abraham E, Wunderink R, Silverman H, Perl TM, Nasraway S, Levy H, et al. Efficacy and safety of monoclonal antibody to human tumor necrosis factor alpha in patients with sepsis syndrome. A randomized, controlled, double-blind, multicenter clinical trial. TNF-alpha MAb Sepsis Study Group. JAMA. 1995;273:934–41.

9. Brochard L, Mancebo J, Wysocki M, Lofaso F, Conti G, Rauss A, et al. Noninvasive Ventilation for Acute Exacerbations of Chronic Obstructive Pulmonary Disease. N Engl J Med. 1995;333:817–22.

10. Sainio V, Kemppainen E, Puolakkainen P, Taavitsainen M, Kivisaari L, Valtonen V, et al. Early antibiotic treatment in acute necrotising pancreatitis. Lancet. 1995;346:663–7.

11. Mangano DT, Layug EL, Wallace A, Tateo I. Effect of Atenolol on Mortality and Cardiovascular Morbidity after Noncardiac Surgery. N Engl J Med. 1996;335:1713–21.

12. Lindner KH, Dirks B, Strohmenger HU, Prengel AW, Lindner IM, Lurie KG. Randomised comparison of epinephrine and vasopressin in patients with out-of-hospital ventricular fibrillation. Lancet. 1997;349:535–7.

13. Umberto Meduri G, Stacey Headley A, Golden E, Carson SJ, Umberger RA, Kelso T, et al. Effect of Prolonged Methylprednisolone Therapy in Unresolving Acute Respiratory Distress Syndrome: A Randomized Controlled Trial. JAMA. 1998;280:159–65.

14. Amato MBP, Barbas CSV, Medeiros DM, Magaldi RB, Schettino GP, Lorenzi-Filho G, et al. Effect of a Protective-Ventilation Strategy on Mortality in the Acute Respiratory Distress Syndrome. N Engl J Med. 1998;338:347–54.

15. Gueugniaud P-Y, Mols P, Goldstein P, Pham E, Dubien P-Y, Deweerdt C, et al. A Comparison of Repeated High Doses and Repeated Standard Doses of Epinephrine for Cardiac Arrest Outside the Hospital. N Engl J Med. 1998;339:1595–601.

16. Kudenchuk PJ, Cobb LA, Copass MK, Cummins RO, Doherty AM, Fahrenbruch CE, et al. Amiodarone for resuscitation after out-of-hospital cardiac arrest due to ventricular fibrillation. N Engl J Med. 1999;341:871–8.

17. Takala J, Ruokonen E, Webster NR, Nielsen MS, Zandstra DF, Vundelinckx G, et al. Increased mortality associated with growth hormone treatment in critically ill adults. N Engl J Med. 1999;341:785–92.

18. Sort P, Navasa M, Arroyo V, Aldeguer X, Planas R, Ruiz-del-Arbol L, et al. Effect of intravenous albumin on renal impairment and mortality in patients with cirrhosis and spontaneous bacterial peritonitis. N Engl J Med. 1999;341:403–9.

19. Sloan EP, Koenigsberg M, Gens D, Cipolle M, Runge J, Mallory MN, et al. Diaspirin cross-linked hemoglobin (DCLHb) in the treatment of severe traumatic hemorrhagic shock: a randomized controlled efficacy trial. JAMA. 1999;282:1857–64.

20. Poldermans D, Boersma E, Bax JJ, Thomson IR, van de Ven LL, Blankensteijn JD, et al. The effect of bisoprolol on perioperative mortality and myocardial infarction in high-risk patients undergoing vascular surgery. Dutch Echocardiographic Cardiac Risk Evaluation Applying Stress Echocardiography Study Group. N Engl J Med. 1999;341:1789–94.

21. Plant PK, Owen JL, Elliott MW. Early use of non-invasive ventilation for acute exacerbations of chronic obstructive pulmonary disease on general respiratory wards: a multicentre randomised controlled trial. Lancet. 2000;355:1931–5.

22. Acute Respiratory Distress Syndrome Network, Brower RG, Matthay MA, Morris A, Schoenfeld D, Thompson BT, et al. Ventilation with lower tidal volumes as compared with traditional tidal volumes for acute lung injury and the acute respiratory distress syndrome. N Engl J Med. 2000;342:1301–8.

23. Nouira S, Marghli S, Belghith M, Besbes L, Elatrous S, Abroug F. Once daily oral ofloxacin in chronic obstructive pulmonary disease exacerbation requiring mechanical ventilation: a randomised placebo-controlled trial. Lancet. 2001;358:2020–5.

24. Warren BL, Eid A, Singer P, Pillay SS, Carl P, Novak I, et al. Caring for the critically ill patient. High-dose antithrombin III in severe sepsis: a randomized controlled trial. JAMA. 2001;286:1869–78.

25. Bernard GR, Vincent JL, Laterre PF, LaRosa SP, Dhainaut JF, Lopez-Rodriguez A, et al. Efficacy and safety of recombinant human activated protein C for severe sepsis. N Engl J Med. 2001;344:699–709.

26. Gattinoni L, Tognoni G, Pesenti A, Taccone P, Mascheroni D, Labarta V, et al. Effect of prone positioning on the survival of patients with acute respiratory failure. N Engl J Med. 2001;345:568–73.

27. O’Grady JG, Burroughs A, Hardy P, Elbourne D, Truesdale A, UK and Republic of Ireland Liver Transplant Study Group. Tacrolimus versus microemulsified ciclosporin in liver transplantation: the TMC randomised controlled trial. Lancet. 2002;360:1119–25.

28. Hypothermia after Cardiac Arrest Study Group. Mild therapeutic hypothermia to improve the neurologic outcome after cardiac arrest. N Engl J Med. 2002;346:549–56.

29. Annane D, Sébille V, Charpentier C, Bollaert P-E, François B, Korach J-M, et al. Effect of treatment with low doses of hydrocortisone and fludrocortisone on mortality in patients with septic shock. JAMA. 2002;288:862–71.

30. Corwin HL, Gettinger A, Pearl RG, Fink MP, Levy MM, Shapiro MJ, et al. Efficacy of recombinant human erythropoietin in critically ill patients: a randomized controlled trial. JAMA. 2002;288:2827–35.

31. Marenzi G, Marana I, Lauri G, Assanelli E, Grazi M, Campodonico J, et al. The prevention of radiocontrast-agent-induced nephropathy by hemofiltration. N Engl J Med. 2003;349:1333–40.

32. Esteban A, Frutos-Vivar F, Ferguson ND, Arabi Y, Apezteguía C, González M, et al. Noninvasive positive-pressure ventilation for respiratory failure after extubation. N Engl J Med. 2004;350:2452–60.

33. Roberts I, Yates D, Sandercock P, Farrell B, Wasserberg J, Lomas G, et al. Effect of intravenous corticosteroids on death within 14 days in 10008 adults with clinically significant head injury (MRC CRASH trial): randomised placebo-controlled trial. Lancet. 2004;364:1321–8.

34. Hallstrom AP, Ornato JP, Weisfeldt M, Travers A, Christenson J, McBurnie MA, et al. Public-access defibrillation and survival after out-of-hospital cardiac arrest. N Engl J Med. 2004;351:637–46.

35. Chen ZM, Jiang LX, Chen YP, Xie JX, Pan HC, Peto R, et al. Addition of clopidogrel to aspirin in 45,852 patients with acute myocardial infarction: randomised placebo-controlled trial. Lancet. 2005;366:1607–21.

36. Steinberg KP, Hudson LD, Goodman RB, Hough CL, Lanken PN, Hyzy R, et al. Efficacy and safety of corticosteroids for persistent acute respiratory distress syndrome. N Engl J Med. 2006;354:1671–84.

37. Hallstrom A, Rea TD, Sayre MR, Christenson J, Anton AR, Mosesso VN Jr, et al. Manual chest compression vs use of an automated chest compression device during resuscitation following out-of-hospital cardiac arrest: a randomized trial. JAMA. 2006;295:2620–8.

38. Corwin HL, Gettinger A, Fabian TC, May A, Pearl RG, Heard S, et al. Efficacy and safety of epoetin alfa in critically ill patients. N Engl J Med. 2007;357:965–76.

39. SAFE Study Investigators, Australian and New Zealand Intensive Care Society Clinical Trials Group, Australian Red Cross Blood Service, George Institute for International Health, Myburgh J, Cooper DJ, et al. Saline or albumin for fluid resuscitation in patients with traumatic brain injury. N Engl J Med. 2007;357:874–84.

40. POISE Study Group, Devereaux PJ, Yang H, Yusuf S, Guyatt G, Leslie K, et al. Effects of extended-release metoprolol succinate in patients undergoing non-cardiac surgery (POISE trial): a randomised controlled trial. Lancet. 2008;371:1839–47.

41. Fergusson DA, Hébert PC, Mazer CD, Fremes S, MacAdams C, Murkin JM, et al. A comparison of aprotinin and lysine analogues in high-risk cardiac surgery. N Engl J Med. 2008;358:2319–31.

42. Girard TD, Kress JP, Fuchs BD, Thomason JWW, Schweickert WD, Pun BT, et al. Efficacy and safety of a paired sedation and ventilator weaning protocol for mechanically ventilated patients in intensive care (Awakening and Breathing Controlled trial): a randomised controlled trial. Lancet. 2008;371:126–34.

43. Russell JA, Walley KR, Singer J, Gordon AC, Hébert PC, Cooper DJ, et al. Vasopressin versus Norepinephrine Infusion in Patients with Septic Shock. N Engl J Med. 2008;358:877–87.

44. Cruz DN, Antonelli M, Fumagalli R, Foltran F, Brienza N, Donati A, et al. Early use of polymyxin B hemoperfusion in abdominal septic shock: the EUPHAS randomized controlled trial. JAMA. 2009;301:2445–52.

45. NICE-SUGAR Study Investigators, Finfer S, Chittock DR, Su SY-S, Blair D, Foster D, et al. Intensive versus conventional glucose control in critically ill patients. N Engl J Med. 2009;360:1283–97.

46. Ferrer M, Sellarés J, Valencia M, Carrillo A, Gonzalez G, Badia JR, et al. Non-invasive ventilation after extubation in hypercapnic patients with chronic respiratory disorders: randomised controlled trial. Lancet. 2009;374:1082–8.

47. CRASH-2 trial collaborators, Shakur H, Roberts I, Bautista R, Caballero J, Coats T, et al. Effects of tranexamic acid on death, vascular occlusive events, and blood transfusion in trauma patients with significant haemorrhage (CRASH-2): a randomised, placebo-controlled trial. Lancet. 2010;376:23–32.

48. De Backer D, Biston P, Devriendt J, Madl C, Chochrad D, Aldecoa C, et al. Comparison of Dopamine and Norepinephrine in the Treatment of Shock. N Engl J Med. 2010;362:779–89.

49. Papazian L, Forel J-M, Gacouin A, Penot-Ragon C, Perrin G, Loundou A, et al. Neuromuscular blockers in early acute respiratory distress syndrome. N Engl J Med. 2010;363:1107–16.

50. Aufderheide TP, Frascone RJ, Wayne MA, Mahoney BD, Swor RA, Domeier RM, et al. Standard cardiopulmonary resuscitation versus active compression-decompression cardiopulmonary resuscitation with augmentation of negative intrathoracic pressure for out-of-hospital cardiac arrest: a randomised trial. Lancet. 2011;377:301–11.

51. Gao Smith F, Perkins GD, Gates S, Young D, McAuley DF, Tunnicliffe W, et al. Effect of intravenous β-2 agonist treatment on clinical outcomes in acute respiratory distress syndrome (BALTI-2): a multicentre, randomised controlled trial. Lancet. 2012;379:229–35.

52. Perner A, Haase N, Guttormsen AB, Tenhunen J, Klemenzson G, Åneman A, et al. Hydroxyethyl starch 130/0.42 versus Ringer’s acetate in severe sepsis. N Engl J Med. 2012;367:124–34.

53. Thielmann M, Kottenberg E, Kleinbongard P, Wendt D, Gedik N, Pasa S, et al. Cardioprotective and prognostic effects of remote ischaemic preconditioning in patients undergoing coronary artery bypass surgery: a single-centre randomised, double-blind, controlled trial. Lancet. 2013;382:597–604.

54. Annane D, Siami S, Jaber S, Martin C, Elatrous S, Declère AD, et al. Effects of fluid resuscitation with colloids vs crystalloids on mortality in critically ill patients presenting with hypovolemic shock: the CRISTAL randomized trial. JAMA. 2013;310:1809–17.

55. Mentzelopoulos SD, Malachias S, Chamos C, Konstantopoulos D, Ntaidou T, Papastylianou A, et al. Vasopressin, steroids, and epinephrine and neurologically favorable survival after in-hospital cardiac arrest: a randomized clinical trial. JAMA. 2013;310:270–9.

56. Guérin C, Reignier J, Richard J-C, Beuret P, Gacouin A, Boulain T, et al. Prone positioning in severe acute respiratory distress syndrome. N Engl J Med. 2013;368:2159–68.

57. Mourvillier B, Tubach F, van de Beek D, Garot D, Pichon N, Georges H, et al. Induced hypothermia in severe bacterial meningitis: a randomized clinical trial. JAMA. 2013;310:2174–83.

58. Ferguson ND, Cook DJ, Guyatt GH, Mehta S, Hand L, Austin P, et al. High-frequency oscillation in early acute respiratory distress syndrome. N Engl J Med. 2013;368:795–805.

59. van Zanten ARH, Sztark F, Kaisers UX, Zielmann S, Felbinger TW, Sablotzki AR, et al. High-protein enteral nutrition enriched with immune-modulating nutrients vs standard high-protein enteral nutrition and nosocomial infections in the ICU: a randomized clinical trial. JAMA. 2014;312:514–24.

60. Amrein K, Schnedl C, Holl A, Riedl R, Christopher KB, Pachler C, et al. Effect of high-dose vitamin D3 on hospital length of stay in critically ill patients with vitamin D deficiency: the VITdAL-ICU randomized clinical trial. JAMA. 2014;312:1520–30.

61. Oostdijk EAN, Kesecioglu J, Schultz MJ, Visser CE, de Jonge E, van Essen EHR, et al. Effects of decontamination of the oropharynx and intestinal tract on antibiotic resistance in ICUs: a randomized clinical trial. JAMA. 2014;312:1429–37.

62. Caironi P, Tognoni G, Masson S, Fumagalli R, Pesenti A, Romero M, et al. Albumin replacement in patients with severe sepsis or septic shock. N Engl J Med. 2014;370:1412–21.

63. Frat J-P, Thille AW, Mercat A, Girault C, Ragot S, Perbet S, et al. High-flow oxygen through nasal cannula in acute hypoxemic respiratory failure. N Engl J Med. 2015;372:2185–96.

64. Murphy GJ, Pike K, Rogers CA, Wordsworth S, Stokes EA, Angelini GD, et al. Liberal or restrictive transfusion after cardiac surgery. N Engl J Med. 2015;372:997–1008.

65. Andrews PJD, Sinclair HL, Rodriguez A, Harris BA, Battison CG, Rhodes JKJ, et al. Hypothermia for Intracranial Hypertension after Traumatic Brain Injury. N Engl J Med. 2015;373:2403–12.

66. Lachman E, Pitsoe SB, Gaffin SL. Anti-lipopolysaccharide immunotherapy in management of septic shock of obstetric and gynaecological origin. Lancet. 1984;1:981–3.

67. Levacher S, Letoumelin P, Pateron D, Blaise M, Lapandry C, Pourriat JL. Early administration of terlipressin plus glyceryl trinitrate to control active upper gastrointestinal bleeding in cirrhotic patients. Lancet. 1995;346:865–8.

68. Feu F, Ruiz del Arbol L, Bañares R, Planas R, Bosch J. Double-blind randomized controlled trial comparing terlipressin and somatostatin for acute variceal hemorrhage. Variceal Bleeding Study Group. Gastroenterology. 1996;111:1291–9.

69. Antonelli M, Conti G, Bufi M, Costa MG, Lappa A, Rocco M, et al. Noninvasive ventilation for treatment of acute respiratory failure in patients undergoing solid organ transplantation: a randomized trial. JAMA. 2000;283:235–41.

70. Lemiale V, Mokart D, Resche-Rigon M, Pène F, Mayaux J, Faucher E, et al. Effect of Noninvasive Ventilation vs Oxygen Therapy on Mortality Among Immunocompromised Patients With Acute Respiratory Failure: A Randomized Clinical Trial. JAMA. 2015;314:1711–9.

71. Hilbert G, Gruson D, Vargas F, Valentino R, Gbikpi-Benissan G, Dupon M, et al. Noninvasive ventilation in immunosuppressed patients with pulmonary infiltrates, fever, and acute respiratory failure. N Engl J Med. 2001;344:481–7.

72. Ronco C, Bellomo R, Homel P, Brendolan A, Dan M, Piccinni P, et al. Effects of different doses in continuous veno-venous haemofiltration on outcomes of acute renal failure: a prospective randomised trial. Lancet. 2000;356:26–30.

73. Palevsky PM, Zhang JH, O’Connor TZ, Chertow GM, Crowley ST, Choudhury D, et al. Intensity of renal support in critically ill patients with acute kidney injury. N Engl J Med. 2008;359:7–20.

74. Bellomo R, Cass A, Cole L, Finfer S, Gallagher M, Lo S, et al. Intensity of continuous renal-replacement therapy in critically ill patients. N Engl J Med. 2009;361:1627–38.

75. Rivers E, Nguyen B, Havstad S, Ressler J, Muzzin A, Knoblich B, et al. Early goal-directed therapy in the treatment of severe sepsis and septic shock. N Engl J Med. 2001;345:1368–77.

76. Goal-Directed Resuscitation for Patients with Early Septic Shock. N Engl J Med. 2014;371:1496–506.

77. Yealy DM, Kellum JA, Huang DT, Barnato AE, Weissfeld LA, Pike F, et al. A randomized trial of protocol-based care for early septic shock. N Engl J Med. 2014;370:1683–93.

78. Mouncey PR, Osborn TM, Power GS, Harrison DA, Sadique MZ, Grieve RD, et al. Trial of early, goal-directed resuscitation for septic shock. N Engl J Med. 2015;372:1301–11.

79. van den Berghe G, Wouters P, Weekers F, Verwaest C, Bruyninckx F, Schetz M, et al. Intensive insulin therapy in critically ill patients. N Engl J Med. 2001;345:1359–67.

80. Finfer S, Chittock DR, Su SY, Blair D, Foster D, Dhingra V, et al. Intensive versus conventional glucose control in critically ill patients. N Engl J Med. 2009;360:1283–97.

81. Dorian P, Cass D, Schwartz B, Cooper R, Gelaznikas R, Barr A. Amiodarone as compared with lidocaine for shock-resistant ventricular fibrillation. N Engl J Med. 2002;346:884–90.

82. Kudenchuk PJ, Brown SP, Daya M, Rea T, Nichol G, Morrison LJ, et al. Amiodarone, Lidocaine, or Placebo in Out-of-Hospital Cardiac Arrest. N Engl J Med. 2016;374:1711–22.

83. de Jonge E, Schultz MJ, Spanjaard L, Bossuyt PM, Vroom MB, Dankert J, et al. Effects of selective decontamination of digestive tract on mortality and acquisition of resistant bacteria in intensive care: a randomised controlled trial. Lancet. 2003;362:1011–6.

84. de Smet AM, Kluytmans JA, Cooper BS, Mascini EM, Benus RF, van der Werf TS, et al. Decontamination of the digestive tract and oropharynx in ICU patients. N Engl J Med. 2009;360:20–31.

85. Myburgh JA, Seppelt IM, Goodman F, Billot L, Correa M, Davis JS, et al. Effect of Selective Decontamination of the Digestive Tract on Hospital Mortality in Critically Ill Patients Receiving Mechanical Ventilation: A Randomized Clinical Trial. JAMA. 2022;328:1911–21.

86. de Silva HA, Fonseka MM, Pathmeswaran A, Alahakone DG, Ratnatilake GA, Gunatilake SB, et al. Multiple-dose activated charcoal for treatment of yellow oleander poisoning: a single-blind, randomised, placebo-controlled trial. Lancet. 2003;361:1935–8.

87. Eddleston M, Juszczak E, Buckley NA, Senarathna L, Mohamed F, Dissanayake W, et al. Multiple-dose activated charcoal in acute self-poisoning: a randomised controlled trial. Lancet. 2008;371:579–87.

88. Olasveengen TM, Sunde K, Brunborg C, Thowsen J, Steen PA, Wik L. Intravenous drug administration during out-of-hospital cardiac arrest: a randomized trial. JAMA. 2009;302:2222–9.

89. Perkins GD, Ji C, Deakin CD, Quinn T, Nolan JP, Scomparin C, et al. A Randomized Trial of Epinephrine in Out-of-Hospital Cardiac Arrest. N Engl J Med. 2018;379:711–21.

90. Morelli A, Ertmer C, Westphal M, Rehberg S, Kampmeier T, Ligges S, et al. Effect of heart rate control with esmolol on hemodynamic and clinical outcomes in patients with septic shock: a randomized clinical trial. JAMA. 2013;310:1683–91.

91. Kakihana Y, Nishida O, Taniguchi T, Okajima M, Morimatsu H, Ogura H, et al. Efficacy and safety of landiolol, an ultra-short-acting β1-selective antagonist, for treatment of sepsis-related tachyarrhythmia (J-Land 3S): a multicentre, open-label, randomised controlled trial. Lancet Respir Med. 2020;8:863–72.

92. Villanueva C, Colomo A, Bosch A, Concepción M, Hernandez-Gea V, Aracil C, et al. Transfusion strategies for acute upper gastrointestinal bleeding. N Engl J Med. 2013;368:11–21.

93. Jairath V, Kahan BC, Gray A, Doré CJ, Mora A, James MW, et al. Restrictive versus liberal blood transfusion for acute upper gastrointestinal bleeding (TRIGGER): a pragmatic, open-label, cluster randomised feasibility trial. Lancet. 2015;386:137–44.

94. Girardis M, Busani S, Damiani E, Donati A, Rinaldi L, Marudi A, et al. Effect of Conservative vs Conventional Oxygen Therapy on Mortality Among Patients in an Intensive Care Unit: The Oxygen-ICU Randomized Clinical Trial. JAMA. 2016;316:1583–9.

95. Schjørring OL, Klitgaard TL, Perner A, Wetterslev J, Lange T, Siegemund M, et al. Lower or Higher Oxygenation Targets for Acute Hypoxemic Respiratory Failure. N Engl J Med. 2021;384:1301–11.

96. Gelissen H, de Grooth HJ, Smulders Y, Wils EJ, de Ruijter W, Vink R, et al. Effect of Low-Normal vs High-Normal Oxygenation Targets on Organ Dysfunction in Critically Ill Patients: A Randomized Clinical Trial. JAMA. 2021;326:940–8.

97. Zarbock A, Kellum JA, Schmidt C, Van Aken H, Wempe C, Pavenstädt H, et al. Effect of Early vs Delayed Initiation of Renal Replacement Therapy on Mortality in Critically Ill Patients With Acute Kidney Injury: The ELAIN Randomized Clinical Trial. JAMA. 2016;315:2190–9.

98. Gaudry S, Hajage D, Schortgen F, Martin-Lefevre L, Pons B, Boulet E, et al. Initiation Strategies for Renal-Replacement Therapy in the Intensive Care Unit. N Engl J Med. 2016;375:122–33.

99. Barbar SD, Clere-Jehl R, Bourredjem A, Hernu R, Montini F, Bruyère R, et al. Timing of Renal-Replacement Therapy in Patients with Acute Kidney Injury and Sepsis. N Engl J Med. 2018;379:1431–42.

100. Timing of Initiation of Renal-Replacement Therapy in Acute Kidney Injury. N Engl J Med. 2020;383:240–51.

101. Shoemaker WC, Appel PL, Kram HB, Waxman K, Lee TS. Prospective trial of supranormal values of survivors as therapeutic goals in high-risk surgical patients. Chest. 1988;94:1176–86.

102. Ulrich C, Harinck-de Weerd JE, Bakker NC, Jacz K, Doornbos L, de Ridder VA. Selective decontamination of the digestive tract with norfloxacin in the prevention of ICU-acquired infections: a prospective randomized study. Intensive Care Med. 1989;15:424–31.

103. Rocha LA, Martín MJ, Pita S, Paz J, Seco C, Margusino L, et al. Prevention of nosocomial infection in critically ill patients by selective decontamination of the digestive tract. A randomized, double blind, placebo-controlled study. Intensive Care Med. 1992;18:398–404.

104. DeRiso AJ 2nd, Ladowski JS, Dillon TA, Justice JW, Peterson AC. Chlorhexidine gluconate 0.12% oral rinse reduces the incidence of total nosocomial respiratory infection and nonprophylactic systemic antibiotic use in patients undergoing heart surgery. Chest. 1996;109:1556–61.

105. Schiffl H, Lang SM, Fischer R. Daily hemodialysis and the outcome of acute renal failure. N Engl J Med. 2002;346:305–10.

106. Lobo SM, Salgado PF, Castillo VG, Borim AA, Polachini CA, Palchetti JC, et al. Effects of maximizing oxygen delivery on morbidity and mortality in high-risk surgical patients. Crit Care Med. 2000;28:3396–404.

107. Auriant I, Jallot A, Hervé P, Cerrina J, Le Roy Ladurie F, Fournier JL, et al. Noninvasive ventilation reduces mortality in acute respiratory failure following lung resection. Am J Respir Crit Care Med. 2001;164:1231–5.

108. Goeters C, Wenn A, Mertes N, Wempe C, Van Aken H, Stehle P, et al. Parenteral L-alanyl-L-glutamine improves 6-month outcome in critically ill patients. Crit Care Med. 2002;30:2032–7.

109. Jacobs S, Price Evans DA, Tariq M, Al Omar NF. Fluconazole improves survival in septic shock: a randomized double-blind prospective study. Crit Care Med. 2003;31:1938–46.

110. Garrel D, Patenaude J, Nedelec B, Samson L, Dorais J, Champoux J, et al. Decreased mortality and infectious morbidity in adult burn patients given enteral glutamine supplements: a prospective, controlled, randomized clinical trial. Crit Care Med. 2003;31:2444–9.

111. Pontes-Arruda A, Aragão AMA, Albuquerque JD. Effects of enteral feeding with eicosapentaenoic acid, gamma-linolenic acid, and antioxidants in mechanically ventilated patients with severe sepsis and septic shock. Crit. Care Med. 2006. p. 2325–33.

112. Fuhrmann JT, Schmeisser A, Schulze MR, Wunderlich C, Schoen SP, Rauwolf T, et al. Levosimendan is superior to enoximone in refractory cardiogenic shock complicating acute myocardial infarction. Crit Care Med. 2008;36:2257–66.

113. Xiao G, Wei J, Yan W, Wang W, Lu Z. Improved outcomes from the administration of progesterone for patients with acute severe traumatic brain injury: a randomized controlled trial. Crit Care. 2008;12:R61.

114. Ornico SR, Lobo SM, Sanches HS, Deberaldini M, Tófoli LT, Vidal AM, et al. Noninvasive ventilation immediately after extubation improves weaning outcome after acute respiratory failure: a randomized controlled trial. Crit Care. 2013;17:R39.

115. Vahedian-Azimi A, Hajiesmaeili M, Amirsavadkouhi A, Jamaati H, Izadi M, Madani SJ, et al. Effect of the Cardio First Angel^TM^ device on CPR indices: a randomized controlled clinical trial. Crit Care. 2016;20:147.
